# Novel Insights into Conformational Rearrangements of the Bacterial Flagellar Switch Complex

**DOI:** 10.1128/mBio.00079-19

**Published:** 2019-04-02

**Authors:** Tomofumi Sakai, Tomoko Miyata, Naoya Terahara, Koichiro Mori, Yumi Inoue, Yusuke V. Morimoto, Takayuki Kato, Keiichi Namba, Tohru Minamino

**Affiliations:** a Graduate School of Frontier Biosciences, Osaka University, Suita, Osaka, Japan; b RIKEN Center for Biosystems Dynamics Research, Suita, Osaka, Japan; c Department of Bioscience and Bioinformatics, Faculty of Computer Science and Systems Engineering, Kyushu Institute of Technology, Iizuka, Fukuoka, Japan; d RIKEN SPring-8 Center, Suita, Osaka, Japan; Cornell University; The Ohio State University

**Keywords:** chemotaxis, flagellar motility, flagellar structure, torque generation

## Abstract

The flagellar motor can spin in both counterclockwise (CCW) and clockwise (CW) directions. The flagellar motor consists of a rotor and multiple stator units, which act as a proton channel. The rotor is composed of the transmembrane MS ring made of FliF and the cytoplasmic C ring consisting of FliG, FliM, and FliN. The C ring is directly involved in rotation and directional switching. The *Salmonella* FliF-FliG deletion fusion motor missing 56 residues from the C terminus of FliF and 94 residues from the N terminus of FliG keeps a domain responsible for the interaction with the stator intact, but its motor function is reduced significantly. Here, we report the structure and function of the FliF-FliG deletion fusion motor. The FliF-FliG deletion fusion not only resulted in a strong CW switch bias but also affected rotor-stator interactions coupled with proton translocation through the proton channel of the stator unit. The energy coupling efficiency of the deletion fusion motor was the same as that of the wild-type motor. Extragenic suppressor mutations in FliG, FliM, or FliN not only relieved the strong CW switch bias but also increased the motor speed at low load. The FliF-FliG deletion fusion made intersubunit interactions between C ring proteins tighter compared to the wild-type motor, whereas the suppressor mutations affect such tighter intersubunit interactions. We propose that a change of intersubunit interactions between the C ring proteins may be required for high-speed motor rotation as well as direction switching.

## Abstract (precis)

The bacterial flagellar motor is a bidirectional rotary motor for motility and chemotaxis, which often plays an important role in infection. The motor is a large transmembrane protein complex composed of a rotor and multiple stator units, which also act as a proton channel. Motor torque is generated through their cyclic association and dissociation coupled with proton translocation through the proton channel. A large cytoplasmic ring of the motor, called C ring, is responsible for rotation and switching by interacting with the stator, but the mechanism remains unknown. By analyzing the structure and function of the wild-type motor and a mutant motor missing part of the C ring connecting itself with the transmembrane rotor ring while keeping a stator-interacting domain for bidirectional torque generation intact, we found interesting clues to the change in the C ring conformation for the switching and rotation involving loose and tight intersubunit interactions.

## INTRODUCTION

The flagellum of Salmonella enterica (hereafter referred to *Salmonella*) is composed of the basal body as a rotary motor, the hook as a universal joint, and the filament as a helical propeller. When the motors rotate counterclockwise (CCW), flagellar filaments form a flagellar bundle behind a cell body to push the cell forward. Quick reversal of motor rotation to clockwise (CW) direction disrupts the flagellar bundle, and so the cell tumbles and changes its swimming direction to migrate toward more favorable environments. The intracellular chemotaxis signaling network modulates flagellar motor switching (1).

**FIG 1:**
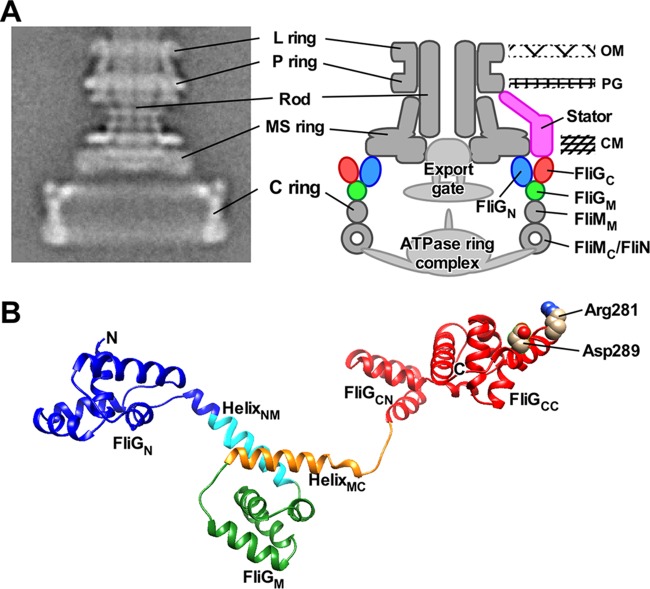
Rotor structure of the flagellar motor. (A) CryoEM image of *Salmonella* basal body (BB) and its schematic diagram. Purified BB consists of the C, MS, L, and P rings and the rod. The type III protein export apparatus, which is composed of a transmembrane export gate complex and a cytoplasmic ATPase complex, and a dozen stator units are associated with the BB. However, these structures are missing during BB purification. The C ring consists of FliG, FliM, and FliN. The N-terminal domain of FliG (FliG_N_) forms the inner lobe structure along with the C-terminal cytoplasmic domain of FliF (FliF_C_). The C-terminal domain of FliG (FliG_C_) is located in the upper part of the C ring. The middle domain of FliM (FliM_M_) is located between the middle domain of FliG (FliG_M_) and FliN and forms a continuous wall of the C ring. A continuous spiral density at the bottom edge of the C ring is made of the C-terminal domain of FliM (FliM_C_) and FliN. (B) Homology model of *Salmonella* FliG. A model was built based on the crystal structure of FliG derived from Aquifex aeolicus (PDB code 3HJL). The Cα backbone is color coded from blue to red, going through the rainbow colors from the N terminus to the C terminus. FliG consists of FliG_N_, FliG_M_, and FliG_C_ domains and two helix linkers named Helix_NM_ and Helix_MC_. FliG_C_ is divided into FliG_CN_ and FliG_CC_ subdomains. Arg281 and Asp289 residues in FliG_CC_ are responsible for interactions with the cytoplasmic domain of the stator unit.

The *Salmonella* flagellar motor is composed of a rotor made of FliF, FliG, FliM, and FliN and a stator consisting of MotA and MotB (Fig. 1A). FliF forms the MS ring in the cytoplasmic membrane. FliG, FliM, and FliN assemble into the C ring on the cytoplasmic face of the MS ring. The C ring is also called the switch complex because it acts as a switch of the direction of flagellar motor rotation. The MotAB complex functions as a transmembrane proton channel to conduct protons to apply force to the rotor. A highly conserved aspartic acid residue of MotB is a proton-binding site in the proton channel, and its protonation and deprotonation are coupled to conformational changes of the cytoplasmic domain of MotA (MotA_C_), which may drive flagellar motor rotation (2, 3). However, the energy coupling mechanism remains unknown.

About 10 stator units exist in a motor at high load, and so the maximum torque produced by the motor is proportional to the number of active stator units in the motor (4, 5). In contrast, a few stator units can drive motor rotation at low load, and so the maximum motor speed depends on the rate of the torque generation cycle of the motor involving stator-rotor interactions coupled with proton translocation through a proton channel (5, 6). The flagellar motor controls the number of active stator units around the rotor but also the proton channel activity of the MotAB complex in response to changes in external load (5, 7).

FliG consists of three domains, FliG_N_, FliG_M_, and FliG_C_, which is further divided into FliG_CN_ and FliG_CC_ subdomains, and two helix linkers, Helix_NM_ and Helix_MC_ (Fig. 1B) (8). FliG_N_ binds to the C-terminal cytoplasmic domain of FliF (FliF_C_) to form an inner lobe structure of the C ring (Fig. 1A) (9–11). Intermolecular FliG_N_-FliG_N_ and FliG_M_-FliG_CN_ interactions promote FliG ring formation (12, 13). The FliMN complex binds to the FliG ring through an interaction between FliG_M_ and the middle domain of FliM (FliM_M_) to form a continuous wall of the C ring (Fig. 1A) (14). FliG_CC_ is located at the most upper part of the wall (Fig. 1A). Two highly conserved charged residues, Arg281 and Asp289, in FliG_CC_ are involved in the interaction with MotA_C_ (Fig. 1B) (15–17).

A chemotaxis signaling protein, CheY-phosphate (CheY-P), binds to FliM and FliN to induce highly cooperative remodeling of the C ring structure, allowing the motor to spin in the CW direction (2). Helix_MC_, which is located at the FliG_M_-FliM_M_ interface, plays an important role in cooperative remodeling of the FliG ring structure responsible for direction switching (18–20). FliG_N_ and Helix_NM_ also contribute to efficient and robust switching (10, 13, 21). However, the switching mechanism remains unclear.

A *Salmonella fliF-fliG* deletion fusion strain (*fliFG_d-f_*) missing the last 56 residues of FliF, which form FliF_C_ and the N-terminal 94 residues of FliG, which form FliG_N_ and an N-terminal portion of Helix_NM_, produces flagella and swims although it has lower motility than the wild type (WT) does (see Fig. S1A in the supplemental material) (9). Electron cryomicroscopy (cryoEM) and image analysis have revealed that a diameter of the C ring produced by the *fliFG_d-f_* mutant is reduced significantly because of a lack of the inner lobe structure (22). However, the C ring wall including FliG_CC_ involved in the interaction with the MotA_C_ looks intact, raising a question of how such a large deletion fusion reduces the motor function. Here, we analyzed the structure and function of the FliF*-*FliG deletion fusion motor (FliFG_d-f_). We show that the FliF*-*FliG deletion fusion causes a strong CW switch bias and that extragenic suppressor mutations in FliG, FliM, or FliN relieve such a strong CW bias. We also show that the FliF*-*FliG deletion fusion reduces the maximum motor speed at low load and that the suppressor mutations increase the maximum speed of the FliFG_d-f_ motor. CryoEM image analysis reveals that the FliF-FliG deletion fusion results in closer intersubunit spacing in the C ring compared to the wild-type motor and that the suppressor mutations affect intersubunit interactions between the C ring proteins.

10.1128/mBio.00079-19.1 FIG S1 Isolation of pseudorevertants from the *fliFG_d-f_* mutant. (A) Motility of strains SJW1103 (WT), SJW3278 (*fliFG_d-f_*), MM3278-8 [*fliFG_d-f _fliG*(D124Y)], MM3278-1 [*fliFG_d-f _fliM*(F188L)], and MM3278-2 [*fliFG_d-f _fliN*(E95G)] in soft agar plates. The plate was incubated at 30°C for 6 h. (B) Fluorescent images of SJW1103 (WT), SJW3278 (*fliFG_d-f_*), and MM3278-1 [*fliFG_d-f _fliM*(F188L)]. The fluorescence images of the filaments labeled with Alexa Fluor 594 (red) were merged with the bright-field images of the cell bodies. (C) Distribution of the number of the flagellar filaments in SJW1103 (WT) (black), SJW3278 (*fliFG_d-f_*) (red), and MM3278-1 [*fliFG_d-f _fliM*(F188L)] (light blue). More than 300 cells for each strain were counted. (D) Measurements of the length of the flagellar filaments. The filament length is the average of more than 100 cells, and vertical lines indicates standard deviations. Download FIG S1, PDF file, 0.5 MB. Copyright © 2019 Sakai et al. 2019 Sakai et al. This content is distributed under the terms of the Creative Commons Attribution 4.0 International license.

## RESULTS

### Effect of the FliF*-*FliG deletion fusion on flagellar formation.

The C ring also acts as a sorting platform for the cytoplasmic ATPase complex of the type III protein export apparatus to facilitate flagellar protein export and assembly (Fig. 1A) (23, 24). To investigate whether the FliF*-*FliG deletion fusion affects flagellar assembly, we labeled flagellar filaments with a fluorescent dye (see Fig. S1B in the supplemental material) and analyzed the number and length of the filaments. The number of filaments produced by wild-type cells ranged from 1 to 8 with an average of 3.4 ± 1.5 (mean ± standard deviation) (Fig. S1C). The average filament length of the wild-type strain was 8.7 ± 1.6 μm (Fig. S1D). In contrast, the *fliFG_d-f_* cells produced filaments ranging from 1 to 8 with an average of 2.7 ± 1.3. This average filament number showed a statistically significant difference compared to that of the wild type (*P < *0.0001) using two-tailed *t* test. However, the average filament length of the *fliFG_d-f_* mutant was 8.4 ± 1.6 μm, which is essentially the same as the wild-type value (Fig. S1D). These results suggest that the FliF*-*FliG deletion fusion affects the assembly of the cytoplasmic ATPase complex into the C ring, thereby reducing the number of flagellar filaments.

### Effect of the FliF*-*FliG deletion fusion on direction switching.

**FIG 2:**
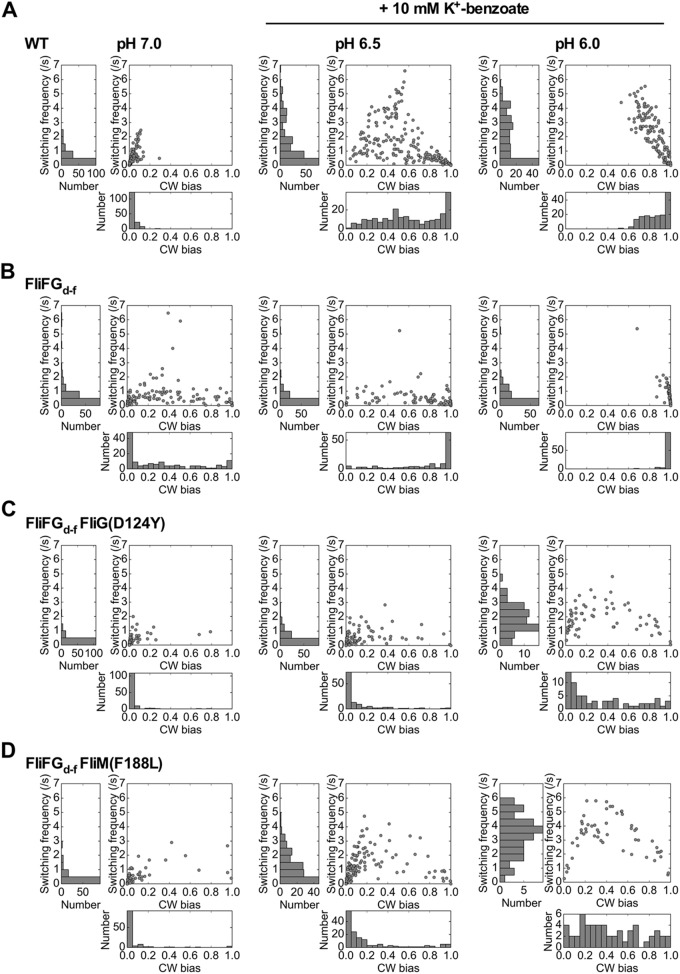
CW bias and switching frequency of the flagellar motor. (A to D) CW bias and switching frequency of wild-type (A), FliFG_d-f_ (B), FliFG_d-f_ FliG(D124Y) (C), and FliFG_d-f_ FliM(F188L) (D) motors were analyzed at pH 7.0 (left panels), at pH 6.5 in the presence of 10 mM potassium benzoate (middle panels), or at pH 6.0 in the presence of 10 mM potassium benzoate (right panels). Rotation measurements of tethered cells were performed at room temperature for 30 s. CW bias versus switching frequency plots were generated from individual motors analyzed under each condition. Histograms of CW bias and switching frequency were shown on the bottom and left, respectively. The number of tethered cells analyzed under each condition are as follows: wild-type cells at pH 7.0, 154 cells; wild-type cells at pH 6.5 with potassium benzoate, 220 cells; wild-type cells at pH 6.0 with potassium benzoate, 156 cells; FliFG_d-f_ at pH 7.0, 136 cells; FliFG_d-f_ at pH 6.5 with potassium benzoate, 127 cells; FliFG_d-f_ at pH 6.0 with potassium benzoate, 106 cells; FliFG_d-f_ FliG(D124Y) at pH 7.0, 116 cells; FliFG_d-f_ FliG(D124Y) at pH 6.5 with potassium benzoate, 121 cells; FliFG_d-f_ FliG(D124Y) at pH 6.0 with potassium benzoate, 67 cells; FliFG_d-f_ FliM(F188L) at pH 7.0, 116 cells; FliFG_d-f_ FliM(F188L) at pH 6.5 with potassium benzoate, 145 cells; FliFG_d-f_ FliG FliM(F188L) at pH 6.0 with potassium benzoate, 55 cells.

It has been reported that swimming motility of the *fliFG_d-f_* mutant is much poorer than that of a wild-type strain presumably due to unusual switching behavior of the flagellar motor (9). To clarify how the FliF-FliG deletion fusion affects the switching function of the flagellar motor, we carried out tethered cell assays to measure the length of CCW and CW rotation intervals. CW bias was calculated as a fraction of the time spent in the CW state of the motor over a 30-s running window. Wild-type tethered cells rotated almost exclusively CCW at external pH 7.0, and so the CW bias of the wild-type motor was 0.03 ± 0.04 (*n* = 154) (Fig. 2A). This suggests that the CheY-P concentration is low under our experimental condition. In contrast, the CW bias of the FliFG_d-f_ motor was 0.32 ± 0.34 (*n* = 136) (Fig. 2B), which is about 10 times higher than that of the wild type. Consistently, about 56% of the *fliFG_d-f_* mutant cells displayed highly tumbly behavior (Fig. S2). These results indicate that the FliF-FliG deletion fusion results in a strong CW switch bias.

10.1128/mBio.00079-19.2 FIG S2 Effect of a FliF-FliG deletion fusion on swimming motility of *Salmonella* cells. (A) Swimming trace of SJW1103 (WT), SJW3278 (*fliFG_d-f_*), MM3278-8 [*fliFG_d-f _fliG*(D124Y)], and MM3278-1 [*fliFG_d-f _fliM*(F188L)] in 1 s. Free swimming of motile cells was observed with a phase contrast microscope. Scale bars indicate 20 μm. (B) Swimming pattern of SJW1103 (WT), SJW3278 (*fliFG_d-f_*), MM3278-8 [*fliFG_d-f _fliG*(D124Y)], and MM3278-1 [*fliFG_d-f _fliM*(F188L)]. Swim, smooth swimming; Zigzag, zigzag motion; Tumble, highly tumbly motility. Free swimming of motile cells were observed with a dark-field microscope. Download FIG S2, PDF file, 0.3 MB. Copyright © 2019 Sakai et al. 2019 Sakai et al. This content is distributed under the terms of the Creative Commons Attribution 4.0 International license.

An in-frame deletion of Pro169-Ala170-Ala171 (PAA) in *Salmonella* FliG locks the motor in the CW state even in the absence of CheY-P (18). To investigate whether the strong CW switch bias of the FliFG_d-f_ motor is dependent on CheY-P, we introduced a *cheY*::Tn*10* allele into the wild-type and *fliFG_d-f_* mutant cells. The wild-type motors rotated only CCW and completely inhibited the switching of motor rotation in the absence of CheY-P (*n* = 20) (Fig. S3A). When we analyzed 30 individual tethered cells of the *fliFG_d-f_ cheY*::Tn*10* mutant, about 80% of the FliFG_d-f_ motors rotated only CCW, whereas the remaining 20% of the motors rotated only CW (Fig. S3B). This suggests that the FliF-FliG deletion fusion causes a mild interference of CCW state of the motor.

10.1128/mBio.00079-19.3 FIG S3 Effect of depletion of CheY-P on CW bias and switching frequency. CW bias and switching frequency of wild-type (A), FliFG_d-f_ (B), FliFG_d-f_ FliG(D124Y) (C), and FliFG_d-f_ FliM(F188L) (D) motors were analyzed in the absence of CheY-P. Rotation measurements of tethered cells with a *cheY*::Tn*10* mutation, which inhibits the switching of rotational direction from CCW to CW, were performed in motility buffer (pH 7.0) at room temperature for 30 s. CW bias versus switching frequency plots of wild-type (A), FliFG_d-f_ (B), FliFG_d-f_ FliG(D124Y) (C), and FliFG_d-f_ FliM(F188L) (D) motors were generated from 20, 30, 20, and 30 individual tethered cells of the *cheY*::Tn*10*, *fliFG_d-f _cheY*::Tn*10*, *fliFG_d-f_ fliG*(D124Y) *cheY*::Tn*10* and *fliFG_d-f_ FliM*(F188L) *cheY*::Tn*10* strains, respectively. Histograms of CW bias and switching frequency were shown on the bottom and left, respectively. Download FIG S3, PDF file, 0.2 MB. Copyright © 2019 Sakai et al. 2019 Sakai et al. This content is distributed under the terms of the Creative Commons Attribution 4.0 International license.

To analyze the sensitivity of the FliFG_d-f_ motor to the chemotactic signal, we carried out tethered cell assays at pH 6.5 in the presence of 10 mM potassium benzoate, which is a repellent that causes tumbling (25). The CW bias and switching frequency of the wild-type motor were increased from 0.03 ± 0.04 and 0.49 ± 0.52 s^−1^ at pH 7.0 (*n* = 154) to 0.61 ± 0.29 and 1.42 ± 1.46 s^−1^, respectively, at pH 6.5 with potassium benzoate (*n* = 220) (Fig. 2A, middle panel). Consistently, no CCW-locked motors were seen. These indicate that the cytoplasmic CheY-P level is increased at pH 6.5 in the presence of potassium benzoate. On the other hand, the CW bias of the FliFG_d-f_ motor was increased from 0.32 ± 0.34 (*n* = 136) to 0.78 ± 0.30 (*n* = 127), whereas the switching frequency of the FliFG_d-f_ motor was decreased from 0.62 ± 0.89 s^−1^ to 0.39 ± 0.60 s^−1^ (Fig. 2B, middle panel). Populations of CW-locked motors were increased significantly in both wild-type and FliFG_d-f_ motors (Fig. 2A and B, left and middle panels). Since a dissociation constant of CheY-P for the CCW motor is estimated to be 4.7-fold higher than that for the CW motor (26), we assume that the switching frequency may be dependent on the dissociation rate of CheY-P from the motor. Therefore, we conclude that the FliF-FliG deletion fusion increases the probability of the CW state independently of CheY-P.

### Effect of the FliF*-*FliG deletion fusion on torque generation.

**FIG 3:**
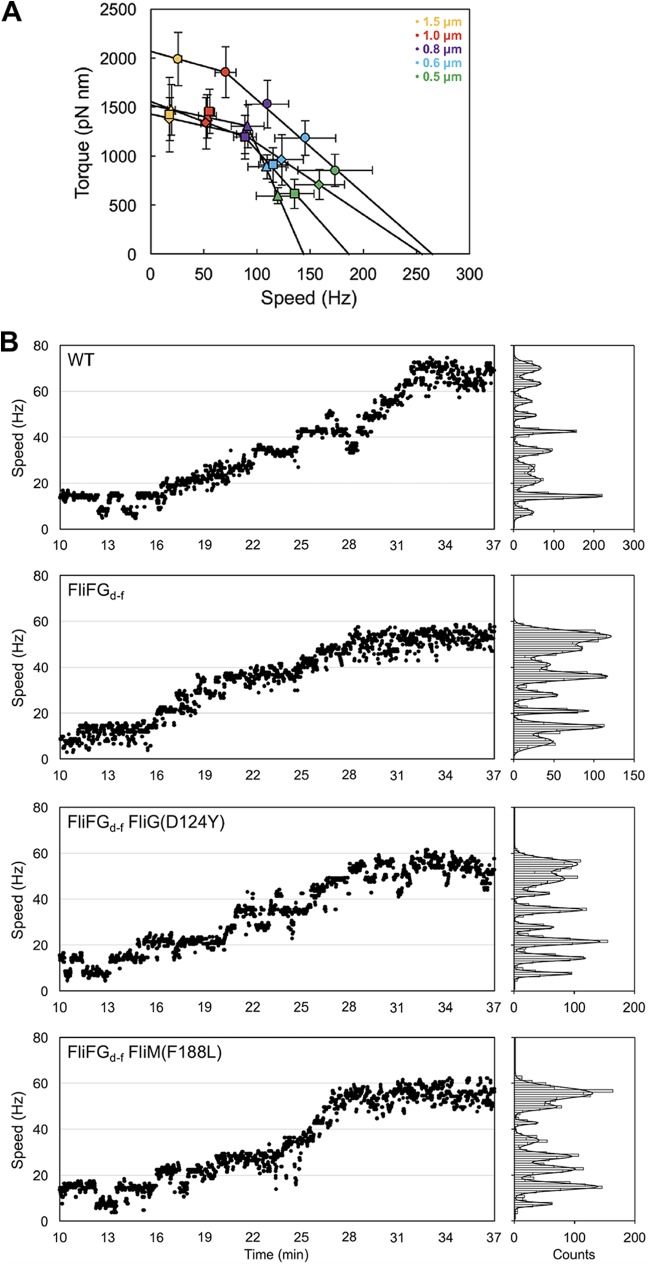
Rotation measurements of the FliFG_d-f_ and its suppressor mutant motors over a wide range of external load. (A) Torque-speed relationship of the wild-type (circles), FliFG_d-f_ (triangles), FliFG_d-f_ FliG(D124Y) (squares), and FliFG_d-f_ FliM(F188L) (diamonds) motors. Rotation measurements were carried out at room temperature by tracking the positions of 1.5-μm (orange), 1.0-μm (red), 0.8-μm (purple), 0.6-μm (cyan), and 0.5-μm (light green) beads attached to the partially sheared sticky filament. Each bead image was recorded for 7 s. (B) Resurrection trace of a single flagellar motor of MM046mAB (indicated as WT), MM3278-46mAB (indicated as FliFG_d-f_), MM3278-8-46mAB [indicated as FliFG_d-f_ FliG(D124Y)], or MM3278-1-46mAB [indicated as FliFG_d-f_ FliM(F188L)] expressing the MotAB complex from an arabinose-inducible promoter on the pBAD24 vector. The cells were grown in L-broth with shaking until the cell density had reached an OD_600_ of ca. 0.6. After incubation with 0.002% arabinose at room temperature for 30 min, rotation measurements were carried out by tracking the positions of 1.0-μm beads attached to the partially sheared sticky filament in a motility buffer containing 0.2% arabinose. The traces and speed histograms are shown on the left and right, respectively. Speed histograms were fitted by multiple Gaussian functions to estimate a unit increment.

To investigate whether the FliF-FliG deletion fusion also affects rotation rates of the flagellar motor over a wide range of external load, bead assays were performed in the absence of CheY-P (Fig. 3A; see also Table S1 in the supplemental material). In agreement with a previous report (27), the maximum torque near stall and the maximum rotational speed near zero load, which were estimated by simple linear extrapolations of the torque-speed curve (Fig. 3A, circles), were 2,068 pN nm and 265 Hz, respectively. The FliFG_d-f_ motor showed a typical torque-speed curve with a gradual decrease in a high-load regime and a rapid drop in a low-load regime in a way similar to the wild-type motor (Fig. 3A, triangles). The maximum torque and rotational speed were estimated to be 1,523 pN nm and 144 Hz, respectively, indicating that the FliF-FliG deletion fusion reduces the maximum torque near stall and the maximum motor speed at zero load.

10.1128/mBio.00079-19.6 TABLE S1 Rotational speed and torque of the flagellar motor. Download Table S1, PDF file, 0.03 MB. Copyright © 2019 Sakai et al. 2019 Sakai et al. This content is distributed under the terms of the Creative Commons Attribution 4.0 International license.

To estimate the average number of active stator units in the FliFG_d-f_ motor at high load, we carried out resurrection experiments. When the MotAB complex was expressed from an arabinose-inducible promoter on a pBAD24-based plasmid, a stepwise increment in rotation rate of a single flagellar motor with a 1.0-μm bead attached was observed (Fig. 3B). The unit increment of the wild-type motor was 7.0 ± 0.7 Hz as judged by multiple Gaussian fitting of speed histograms (Fig. 3B). Each increment unit reflects the incorporation of a single stator unit around the rotor at high load (4). Because the average speed of the wild-type motor with a 1.0-μm bead attached was 71 ± 10 Hz (Table S1), the average number of active stator units was estimated to be about 10 in the wild-type motor, in agreement with a previous report (28). Similar stepwise increments were observed in the FliFG_d-f_ motor, and the increment unit was 6.8 ± 0.9 Hz (Fig. 3B), indicating that the energy coupling efficiency of the FliFG_d-f_ motor is essentially the same as that of the wild-type motor. Since the average speed of the FliFG_d-f_ motor with a 1.0-μm bead attached was about 54 ± 8 Hz (Table S1), the average number of active stator units was estimated to be about eight in the FliFG_d-f_ motor. The maximum rotation speed of the motor at low load is limited by the rate of torque generation cycle of the motor (6). Since the FliF-FliG deletion fusion did not affect the energy coupling efficiency at all, we suggest that a considerable reduction in the maximum speed of the FliFG_d-f_ motor presumably results from a decrease in the rate of conformational dynamics in the interactions between MotA_C_ and FliG_CC_ coupled with the proton flow through the MotAB complex.

**FIG 4:**
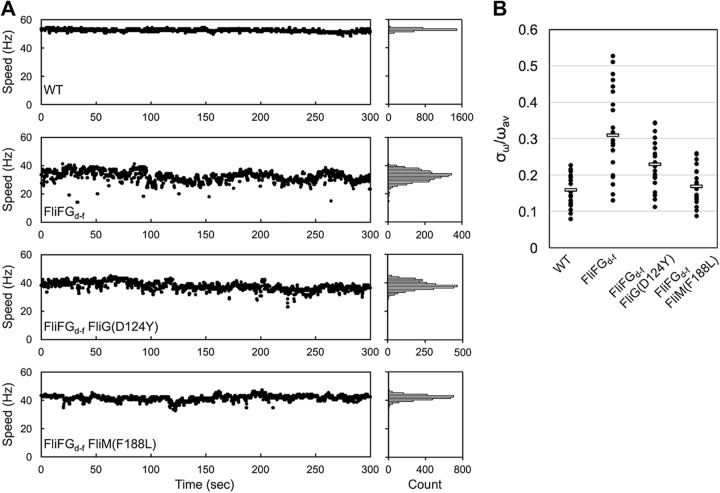
Effect of the FliF-FliG deletion fusion on speed stability of flagellar motor rotation. (A) Typical examples of motor speed versus time plots of the wild-type, FliFG_d-f_ , FliFG_d-f_ FliG(D124Y), and FliFG_d-f_ FliM(F188L) motors. Rotation measurements of 1.0-μm beads attached to the wild-type, FliFG_d-f_, FliFG_d-f_ FliG(D124Y), and FliFG_d-f_ FliM(F188L) motors were carried out at room temperature for 300 s. Speed histograms are shown on the left of the traces. (B) Speed fluctuation of the wild-type, FliFG_d-f_, FliFG_d-f_ FliG(D124Y), and FliFG_d-f_ FliM(F188L) motors. The values of the average speeds (ω_av_) and their standard deviations (σ_ω_) were calculated. Black dots indicate individual motors.

Figure 4A shows typical examples of bead rotation attached to the wild-type and FliFG_d-f_ motors. The rotation rates of the FliFG_d-f_ motors with 1.0-μm beads attached largely fluctuated over a 300-s running window, whereas the wild-type motors did not. To quantitatively evaluate the speed stability of flagellar motor rotation, we calculated the ratio of standard deviation (σ_ω_) and average of the rotation speed (ω_av_) (Fig. 4B and Table S2). The value of σ_ω_/ω_av_ was 0.16 ± 0.04 for the wild-type motor (*n* = 20) and 0.31 ± 0.12 for the FliFG_d-f_ motor (*n* = 20). This suggests that the FliFG_d-f_ motor cannot produce torque constantly.

10.1128/mBio.00079-19.7 TABLE S2 Speed fluctuations of the flagellar motor. Download Table S2, PDF file, 0.02 MB. Copyright © 2019 Sakai et al. 2019 Sakai et al. This content is distributed under the terms of the Creative Commons Attribution 4.0 International license.

### Isolation of pseudorevertants from the *fliFG_d-f_* mutant.

**FIG 5:**
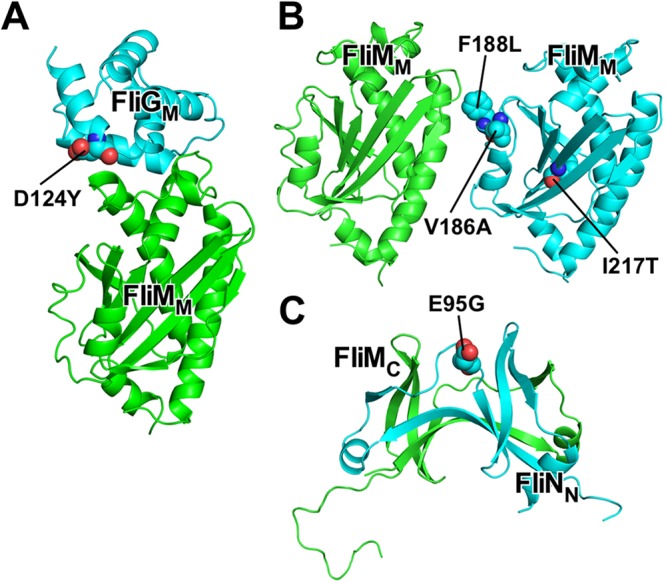
Locations of suppressor mutations of a FliF-FliG deletion fusion. (A) Homology model of *Salmonella* FliG_M_-FliM_M_ complex and location of the FliG(D124Y) suppressor mutation. A homology model was built based on the crystal structure of Thermotoga maritima FliG_M_-FliM_M_ complex (PDB code 3SOH). Cα ribbon drawing of FliG_M_ (cyan) and FliM_M_ (green). (B) Model of FliM subunit arrangement in the C ring and locations of the FliM(V186A), FliM(F188L), and FliM(I217T) suppressor mutations. A homology model of *Salmonella* FliM_M_ was built based on the crystal structure of Thermotoga maritima FliM_M_ (PDB code 3SOH). (C) Crystal structure of *Salmonella* FliM_C_-FliN_N_ fusion protein (PDB code 4YXB) and location of the FliN(E95G) suppressor mutation. FliM_C_ and FliN_N_ subunits are shown by green and cyan Cα ribbon models, respectively.

To clarify how the FliF-FliG deletion fusion affects the motor function, we isolated 13 pseudorevertants from the *fliFG_d-f_* mutant (Fig. S1A). These suppressor mutations did not improve filament formation at all (Fig. S1B, C, and D), indicating that they significantly restore the function of the FliFG_d-f_ motor. DNA sequence analysis identified the D124Y mutation in FliG, the V186A, F188L (isolated nine times), and I217T mutations in FliM, and the E95G mutation in FliN. The FliG(D124Y) mutation is located at the FliG_M_-FliM_M_ interface (Fig. 5A). The FliM(V186A) and FliM(F188L) mutations are located at an interface between FliM subunits (14), whereas the FliM(I217T) mutation lies in a hydrophobic core of FliM (Fig. 5B). The FliN(E95G) mutation is located at an interface between FliM and FliN (Fig. 5C). These results suggest that these suppressor mutations affect intersubunit interactions between the C ring proteins.

### Effect of the FliG(D124Y) and FliM(F188L) mutations on motor performance of the FliFG_d-f_ motor.

To test whether suppressor mutations in FliG, FliM, or FliN relieve a strong CW switch bias of the FliFG_d-f_ motor, we selected two suppressor mutant motors, FliFG_d-f_ FliG(D124Y) and FliFG_d-f_ FliM(F188L) and analyzed their CW bias at pH 7.0 (Fig. 2C and D, left panels). In contrast to the FliFG_d-f_ motor, of which CW bias was 0.32, the mean CW bias values of the FliFG_d-f_ FliG(D124Y) and FliFG_d-f_ FliM(F188L) motors were 0.03 and 0.06, respectively, which are almost the same as the wild-type value (0.03 for the wild-type motor). This indicates that the FliG(D124Y) and FliM(F188L) mutations relieve the strong CW bias caused by the deletion fusion. However, a very small fraction of the FliFG_d-f_ FliG(D124Y) and FliFG_d-f_ FliM(F188L) motors remained to rotate CW in the absence of CheY-P (Fig. S3C and D). To investigate how they relieve the strong CW bias caused by the FliF-FliG deletion fusion, we analyzed the CW bias of the FliFG_d-f_ FliG(D124Y) and FliFG_d-f_ FliM(F188L) motors at pH 6.5 in the presence of 10 mM potassium benzoate (Fig. 2C and D, middle panels). In contrast to the wild-type motor, a very large fraction of these two mutant tethered cells remained to rotate exclusively CCW, indicating that the FliG(D124Y) and FliM(F188L) mutations result in a strong CCW bias. We further decreased the external pH from 6.5 to 6.0 in the presence of 10 mM potassium benzoate and measured the CW bias of the FliFG_d-f_ FliG(D124Y) and FliFG_d-f_ FliM(F188L) motors. The CW bias and switching frequency of the wild-type and FliFG_d-f_ motors were 0.86 ± 0.12 and 1.83 ± 1.58 s^−1^ (*n* = 156) and 0.98 ± 0.04 and 0.57 ± 0.72 s^−1^ (*n* = 106), respectively (Fig. 2A and B, right panels). In contrast, the CW bias and switching frequency of the FliFG_d-f_ FliG(D124Y) and FliFG_d-f_ FliM(F188L) motors were 0.34 ± 0.32 and 1.85 ± 0.96 s^−1^ (*n* = 67) and 0.43 ± 0.28 and 3.29 ± 1.41 s^−1^ (*n* = 106), respectively (Fig. 2C and D, right panels). These results suggest that the FliG(D124Y) and FliM(F188L) mutations stabilize the CCW conformation of the FliFG_d-f_ motor.

We investigated whether the FliG(D124Y) and FliM(F188L) mutations also improve the torque generation process of the FliFG_d-f_ motor. The maximum torques of the FliFG_d-f_ FliG(D124Y) and FliFG_d-f_ FliM(F188L) motors were estimated to be 1,561 pN nm and 1,430 pN nm, respectively, which were almost the same as that of the FliFG_d-f_ motor (1,523 pN nm) (Fig. 3A and Table S1). Resurrection experiments revealed that the increment units of the FliFG_d-f_ FliG(D124Y) and FliFG_d-f_ FliM(F188L) motors were 6.9 ± 0.8 Hz and 6.9 ± 0.8 Hz, respectively, which are essentially the same as those of the wild-type and FliFG_d-f_ motors (Fig. 3B). In contrast, the maximum rotational speeds of the FliFG_d-f_ FliG(D124Y) and FliFG_d-f_ FliM(F188L) motors, which were estimated by a simple linear extrapolation of their torque-speed curves, were 187 Hz and 256 Hz, respectively (Fig. 3A), which are higher than that of the FliFG_d-f_ motor (144 Hz.) This suggests that these suppressor mutations increase the rate of the mechanochemical reaction cycle of the FliFG_d-f_ motor.

To test whether the FliG(D124Y) and FliM(F188L) mutations suppress the speed fluctuation of the FliFG_d-f_ motor, we calculated σ_ω_ and ω_av_ of the FliFG_d-f_ FliG(D124Y) and FliFG_d-f_ FliM(F188L) motors (Table S2). The values of σ_ω_/ω_av_ were 0.23 ± 0.07 for the FliFG_d-f_ FliG(D124Y) motor (*n* = 20) and 0.17 ± 0.05 for the FliFG_d-f_ FliM(F188L) motor (*n* = 20) (Fig. 4B and Table S2), indicating that these suppressor mutations significantly stabilize the rotation of the FliFG_d-f_ motor.

### Effect of the FliG(D124Y) mutation on the C ring structure of the FliFGd-f motor.

**FIG 6:**
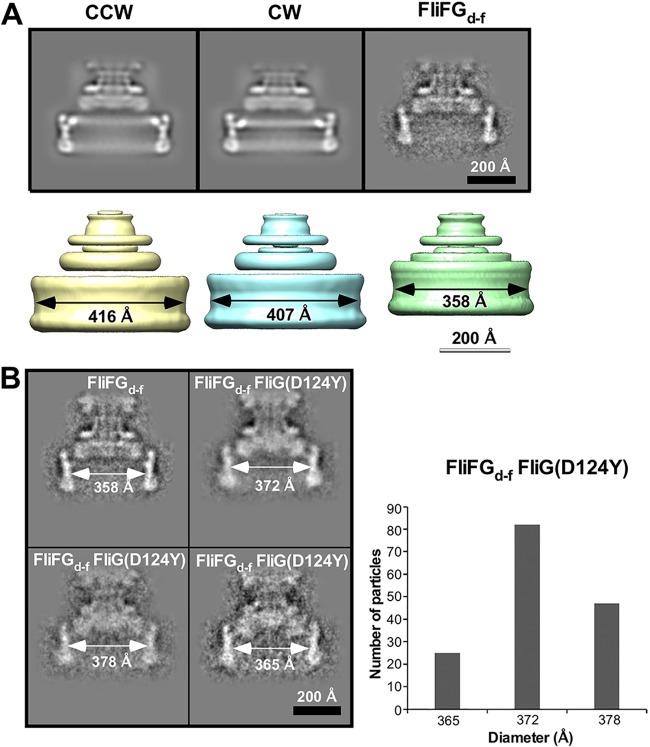
CryoEM image analysis of the C ring structure. (A) Projection images (top) and 3D volume maps (bottom) of the MS-C ring complex of the CCW, CW, and FliFG_d-f_ motors with C34, C34, and C31 symmetries, respectively. (B) Side views of representative 2D class averages of the MS-C ring complex of the FliFG_d-f_ FliG(D124Y) motor. A histogram of the C ring diameter is shown on the right.

To clarify structural differences between the FliFG_d-f_ motor and its suppressor mutant motors, we carried out cryoEM image analysis. We isolated hook-basal bodies (HBBs) and basal bodies (BBs) from the wild-type and *fliG*(ΔPAA) strains, of which motors are put in the default CCW and CW states, respectively, to use the CCW and CW motors as the controls. In agreement with previous reports (29, 30), the rotational symmetry of the C rings of the CCW and CW motors varied from 32-fold to 35-fold, and the diameter of the ring showed a similar range of variability (Fig. S4). Because 14,417 of 30,655 C rings of the CCW motor and 9,201 of 23,163 C rings of the CW motor were assigned to C34 symmetry, we used cryoEM images of the C ring with C34 symmetry and reconstructed the 3D images of the C ring in the CCW and CW states (Fig. 6A). The C ring diameters of the CCW and CW motors were 416 Å and 407 Å, respectively, and so the unit repeat distances along the circumference of the C ring were calculated to be 38.4 Å and 37.6 Å for the CCW and CW motors, respectively. This indicates that the component proteins of the C ring structure, FliG, FliM, and FliN, are more densely packed in the CW state than in the CCW state. The rotational symmetry of the C ring of the FliFG_d-f_ motor ranged from 28-fold to 33-fold (Fig. S4), with the subunit number smaller than the wild-type motor by 2 to 4. Because 910 of 2,301 C rings of the FliFG_d-f_ motor were assigned to C31 symmetry, we used cryoEM images of the C ring with C31 symmetry to reconstruct the 3D image (Fig. 6A). In agreement with a previous report (22), the inner lobe structure was missing from the C ring. The diameter of the C ring was measured to be 358 Å, and so the unit repeat distance was calculated to be 36.3 Å, indicating that the intersubunit spacing in the C ring of the FliFG_d-f_ motor becomes lower compared to the wild-type motor.

10.1128/mBio.00079-19.4 FIG S4 Stoichiometry of the C ring. (A) End-on view of representative reference-free 2D class average images of the C ring derived from the CCW (top panels), CW (middle panels), and FliFG_d-f_ motors. (B) Histogram of the number of particles with distinct rotational symmetries. Download FIG S4, PDF file, 1.1 MB. Copyright © 2019 Sakai et al. 2019 Sakai et al. This content is distributed under the terms of the Creative Commons Attribution 4.0 International license.

To test whether the suppressor mutations in FliG, FliM, and FliN affect the intersubunit spacing in the C ring, we isolated HBBs from the pseudorevertants of the *fliFG_d-f_* mutant. However, the C ring structure was very fragile and was easily dissociated from the HBB during purification, suggesting that the suppressor mutations weaken intersubunit interactions between FliG, FliM, and FliN in the C ring. We were able to obtain only a very small fraction of the HBBs with the C ring attached from the *fliFG_d-f_ fliG*(D124Y) mutant and so carried out 2D classification of the C ring structure. Using typical 2D images, we built the 3D model with C100 symmetry to measure a diameter of the C ring. The diameter of the C ring of 82 motors out of 154 FliFG_d-f_ FliG(D124Y) motors was measured to be 372 Å (Fig. 6B). Both CCW- and CW-stated wild-type motors have C34 symmetry, raising the possibility that the C ring of the FliFG_d-f_ FliG(D124Y) motor may have C31 symmetry in a way similar to the FliFG_d-f_ motor. If true, the unit repeat distance was estimated to be 37.7 Å, which is 1.4 Å longer than that of the FliFG_d-f_ motor. Since the FliG(D124Y) mutation is located at the FliG_M_-FliM_M_ interface (Fig. 5A), we suggest that the FliG(D124Y) mutation may affect intersubunit interactions between C ring component proteins in the FliFG_d-f_ motor, thereby increasing the C ring diameter. However, it is also possible that the FliG(D124Y) mutation may increase the rotational symmetry from C31 to C32, thereby increasing a diameter of the C ring from 358 Å to 372 Å.

## DISCUSSION

A FliF-FliG full-length fusion results in a strong CW switch bias, thereby causing highly tumbly behavior of Escherichia coli cells (13). Suppressor mutations, which restore the chemotactic defect of the E. coli *fliF-fliG* full-length fusion strain, are located at the FliG_N_-FliG_N_ interface, suggesting that the FliG_N_-FliG_N_ interaction contributes to efficient and robust flagellar motor switching (13). Here, we analyzed the motor performance of the FliFG_d-f_ motor missing FliF_C_ and FliG_N_. The FliF-FliG deletion fusion resulted in a strong CW switch bias (Fig. 2). Interestingly, about 20% of the FliFG_d-f_ motors remained in the CW state even in the absence of CheY-P (see Fig. S3 in the supplemental material), indicating that the FliF-FliG deletion fusion locks the motor in the CW state. Therefore, we suggest that the FliF-FliG deletion fusion strongly biases the C ring structure toward the CW conformation.

To clarify a structural difference between the CCW and CW motors, we performed cryoEM image analyses and found that the unit repeat distances of the C ring with C34 symmetry were estimated to be 38.4 Å and 37.6 Å for the CCW and CW motors, respectively (Fig. 6A). This indicates that the C ring structure is more densely packed in the CW state than in the CCW state. The FliF-FliG deletion fusion made intersubunit spacing between C ring component proteins lower than the spacing in the wild-type motor (Fig. 6A). Therefore, we propose that intersubunit interactions between the C ring proteins switch from a loose packing mode to a tight one when directional switching occurs.

Why does the FliF-FliG deletion fusion stabilize the CW state of the motor? A conformational change of Helix_MC_ connecting FliG_M_ and FliG_C_ is postulated to be responsible for directional switching (18, 19). The E95D, D96V/Y, T103S, G106A/C, and E108K substitutions and in-frame deletion of residues 87 to 96 in Helix_NM_ of FliG result in a strong CW switch bias (10, 21). Interestingly, Thr103 in Helix_NM_ directly makes hydrophobic contacts with Pro169 and Ala173 in Helix_MC_ (Fig. S5). Therefore, we propose that the FliF-FliG deletion fusion may affect the Helix_NM_-Helix_MC_ interaction to allow Helix_MC_ to adopt the CW conformation. The FliG(D124Y) suppressor mutation is located at an interface between FliG_M_ and FliM_M_ (Fig. 5A) and results in the strong CCW switch bias (Fig. 2). Since Helix_MC_ makes hydrophobic contacts with FliG_M_ and FliM_M_ (14), we propose that this FliG mutation may stabilize the CCW conformation of Helix_MC_.

10.1128/mBio.00079-19.5 FIG S5 Interaction between Helix_NM_ and Helix_MC_. Cα ribbon drawing of Helix_NM_ (green) and Helix_MC_ of *Salmonella* FliG homology model. The E95D, D96V/Y, T103S, G106A/C, and E108K substitutions in Helix_NM_ of FliG result in a strong CW switch bias. An in-frame deletion of Pro169, Ala170, and Ala171 confers a CW-locked phenotype. Thr103 makes hydrophobic contacts with Pro169 and Ala173 residues. Download FIG S5, PDF file, 0.1 MB. Copyright © 2019 Sakai et al. 2019 Sakai et al. This content is distributed under the terms of the Creative Commons Attribution 4.0 International license.

A dozen stator units work in the motor at high load, whereas only a few stators do at low load (5). Here, we found that the average number of active stator units was two units less in the FliFG_d-f_ motor than in the wild-type motor at high load (Fig. 3). CryoEM image analyses revealed that the diameter of the C ring of the FliFG_d-f_ motor was smaller than that of the wild-type motor (Fig. 6A), supporting a plausible hypothesis that the diameter of the C ring determines the structural capacity for the number of active stator units to bind to the motor (31). Resurrection experiments revealed that the FliF-FliG deletion fusion did not affect the energy coupling efficiency of the motor (Fig. 3B). In contrast to the wild-type motor, the rotational speed of the FliFG_d-f_ motor largely fluctuated at high load (Fig. 4), indicating that the FliFG_d-f_ motor cannot produce torque constantly. This suggests that the FliF-FliG deletion fusion may affect the positioning and orientation of FliG_CC_ at the stator-rotor interface during motor rotation, causing large speed fluctuations. The FliG(D124Y) and FliM(F188L) mutations suppressed such speed fluctuations significantly (Fig. 4). Therefore, we propose that these mutations may allow FliG_CC_ to adjust its alignment relative to the MotAB complex to generate torque constantly. Since the motor speed at high load is proportional to the number of active stator units in the motor (4), it is also possible that the speed fluctuation of the FliFG_d-f_ motor presumably results from rapid increase and decrease in the number of functionally active stator units in the motor during motor rotation.

The motor speed at low load depends on the rate of the mechanochemical reaction cycle of the motor (6). We showed that the maximum speed of the FliFG_d-f_ motor near zero load was lower than that of the wild-type motor (Fig. 3A). This suggests that the FliF-FliG deletion fusion restricts conformational dynamics of MotA_C_ coupled with the proton flow through the MotAB proton channel at low load. The FliG(D124Y) and FliM(F188L) mutations increased the maximum motor speed from 144 Hz to 187 Hz and 256 Hz, respectively (Fig. 4A). This indicates that a conformational change of the C ring must have occurred when the motor speed was increased by these mutations. CryoEM image analysis revealed that the FliG(D124Y) mutation induces a conformational change in the C ring of the FliFG_d-f_ motor (Fig. 6B), Therefore, we propose that a change of intersubunit interactions between the C ring proteins may allow the C ring to adopt a certain conformation suitable for high-speed motor rotation at low load.

## MATERIALS AND METHODS

### Bacterial strains and media.

Bacterial strains and plasmids are listed in Table S3 in the supplemental material. L-broth, T-broth, and soft agar plates were prepared as described previously (32–34).

10.1128/mBio.00079-19.8 TABLE S3 Strains and plasmids used in this study. Download Table S3, PDF file, 0.05 MB. Copyright © 2019 Sakai et al. 2019 Sakai et al. This content is distributed under the terms of the Creative Commons Attribution 4.0 International license.

### P22-mediated transduction and DNA sequencing.

P22-mediated transduction was performed as described previously (35). DNA sequencing reactions were conducted using BigDye v3.1 (Applied Biosystems), and then the reaction mixtures were analyzed by a 3130 genetic analyzer (Applied Biosystems).

### Motility assays.

Fresh colonies were inoculated onto soft agar plates and incubated at 30°C. Swimming behavior of *Salmonella* cells was observed in motility buffer (10 mM potassium phosphate, 0.1 mM EDTA, 10 mM sodium lactate; pH 7.0) by phase-contrast microscopy as described previously (36). Flagellar filaments of *Salmonella* cells swimming in the motility buffer were observed by dark field microscopy as described previously (37).

### Fluorescent staining of flagellar filaments.

Flagellar filaments were labeled with polyclonal anti-FliC antibody and anti-rabbit IgG conjugated with Alexa Fluor 594 (Invitrogen) and were observed by fluorescence microscopy as described previously (38). Fluorescence images were processed using ImageJ software version 1.51 (National Institutes of Health). Statistical analyses were done using the StatPlus::mac software (AnalystSoft), and comparisons were performed using a two-tailed Student’s *t* test.

### Tethered cell assays.

*Salmonella* cells producing sticky flagellar filaments, of which component protein (flagellin, FliC) lacks residues 205 to 293 (39), were grown overnight in T-broth at 30°C. The cells were inoculated into fresh T-broth, followed by incubation at 30°C with shaking for 3.5 h. A 0.5-ml culture was passed through a 25-gauge needle to partially shear sticky flagellar filaments off. The cells were attached to a glass surface. After the cells were washed twice with motility buffer, tethered cell rotation was observed under an inverted phase-contrast microscope (IX 71; Olympus) with a 60× oil immersion phase difference lens objective (PlanApo 60×/1.4 infinity/Olympus). The rotation was recorded by a high-speed CCD camera (Digimo-VCC-SXGA-B; Digimo) at 100 frames per second. To calculate CW bias, which is defined as a fraction of the time spent in the CW state over a 30-s running window recording, motor switching traces were converted to binary traces by assigning positive and negative speeds as described by Lele and Berg (40), and CW bias and switching frequency were calculated.

### Bead assays.

Bead assays are performed at room temperature as described previously (41). Polystyrene beads with a diameter of 1.5, 1.0, 0.8, 0.6, or 0.5 μm (Invitrogen) were used in this study. Each bead image was recorded by a high-speed CCD camera at 1,000 or 2,000 frames per second. Resurrection experiments were conducted as described previously (42). Rotation rate, rotation radius, and torque were calculated as described previously (41). Each rotation rate is the average of 7 s of data.

To evaluate speed stability of the flagellar motor, the average rotation rate, ω_av_, and standard deviation, σ_ω_, of each rotation data was calculated as described before (7). The values of ω_av_ and σ_ω_ were obtained by tracking the positions of 1.0-μm beads for 300 s.

### CryoEM image analysis.

HBBs and BBs with the C ring attached were prepared from *Salmonella* cells as described previously (43). A 3-μl solution of HBBs or BBs was applied to a holey carbon grid (Quantifoil R0.6/1; Quantifoil Micro Tools), which had been glow discharged in a weak vacuum for 5 s immediately before use. The grids were blotted twice for 3 s with a 1-s drain time and were quickly frozen in liquid ethane using Vitrobot (FEI). CryoEM images were collected by a JEM-3200FSC electron microscope (JEOL) equipped with a liquid-nitrogen-cooled specimen stage, an Ω-type energy filter, and a field-emission electron gun, operated at an accelerating voltage of 200 kV. The images were captured by a F415mp CCD camera (TVIPS) at a magnification of ×88,800 corresponding to a pixel size of 1.69 Å or a F816 CCD camera (TVIPS) at a magnification of ×66,300 corresponding to a pixel size of 2.35 Å, a defocus range of 1.0 to 2.5 μm, and an electron dose of 40 e^−^ per Å^2^. Defocus and astigmatism in the cryoEM images were determined using CTFFIND3 (44).

To estimate the symmetry of the C ring, end-on view images of BBs were boxed out by BOXER (45). Each end-on view image was converted from Cartesian to polar coordinates, and then the autocorrelation function was calculated. Rotational symmetry was analyzed from Fourier transformation of the autocorrelation function. The end-on view images with distinct rotational symmetries were classified, aligned, and averaged.

To measure the diameter of the C ring with the most popular symmetry, 3D reconstruction was performed using cryoEM images of the HBBs boxed out by Boxer (45). Since rotational symmetries were different among the C ring images we obtained, the C rings with the most popular symmetries were selected from the HBBs isolated from the wild-type (CCW motor), *fliG*(ΔPAA) (CW motor), and *fliFG_d-f_* (FliFG_d-f_ motor) strains by 3D classification. For the 3D classification, three initial models (for CCW and CW motors) and two initial modesl (for FliFG_d-f_ motor) with a radius measured from end-on view were prepared. The 3D classifications were done by MULTIREFINE applied with C100 symmetry. Then, reprojection from the end-on view was calculated. Each diameter was defined by measuring from a center to the highest density position in the reprojection. Since the total cryoEM image number of the FliFG_d-f_ FliG(D124Y) motor was not enough to run the MULTIREFINE, the 3D structure was reconstructed by MAKE3D with C100 symmetry using a typical 2D average image obtained by the REFINE2D.PY program to measure the C ring diameter.
