# Supplementary figures and images for: Novel Insights into Conformational Rearrangements of the Bacterial Flagellar Switch Complex
Source: mBio. 2019 Apr 2;10(2):e00079-19. doi: 10.1128/mBio.00079-19 (PMC6445934; doi:10.1128/mBio.00079-19)

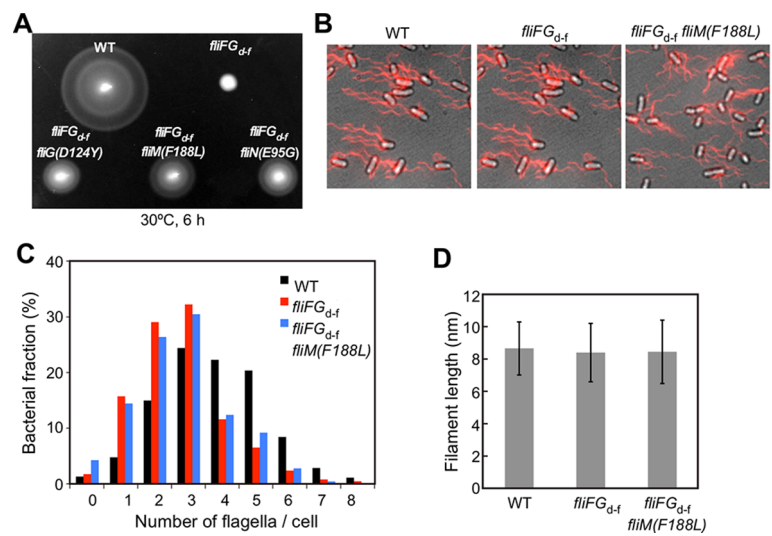

Supplement: FIG S1 [file mBio.00079-19-sf001.pdf]

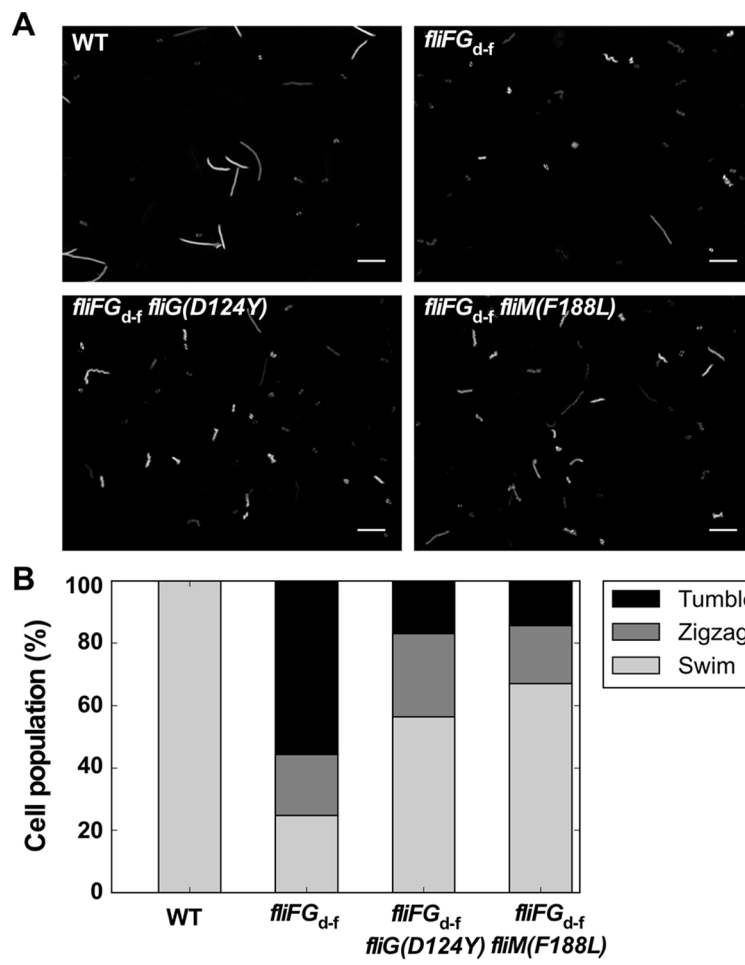

Supplement: FIG S2 [file mBio.00079-19-sf002.pdf]

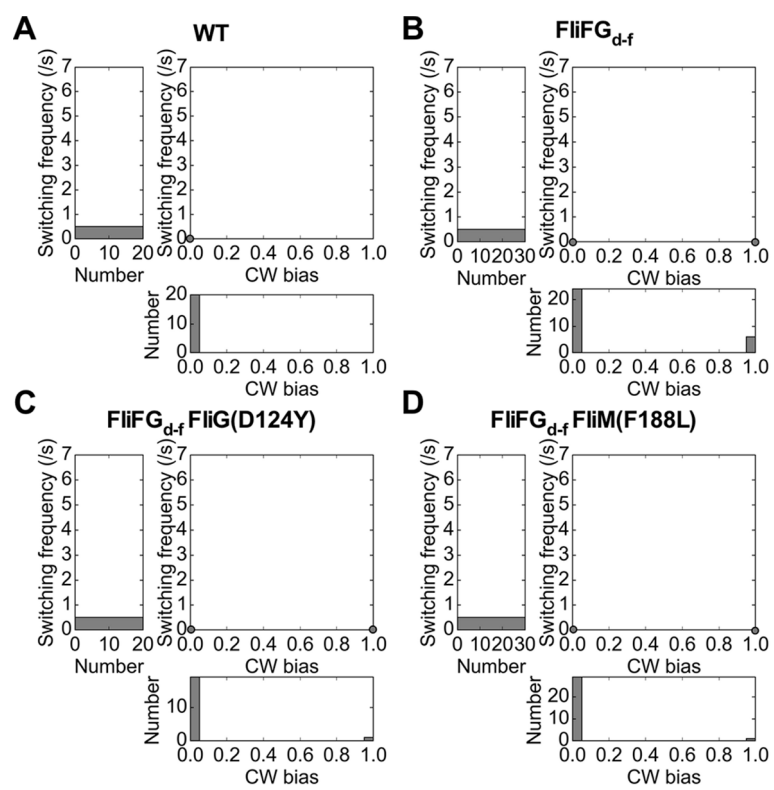

Supplement: FIG S3 [file mBio.00079-19-sf003.pdf]

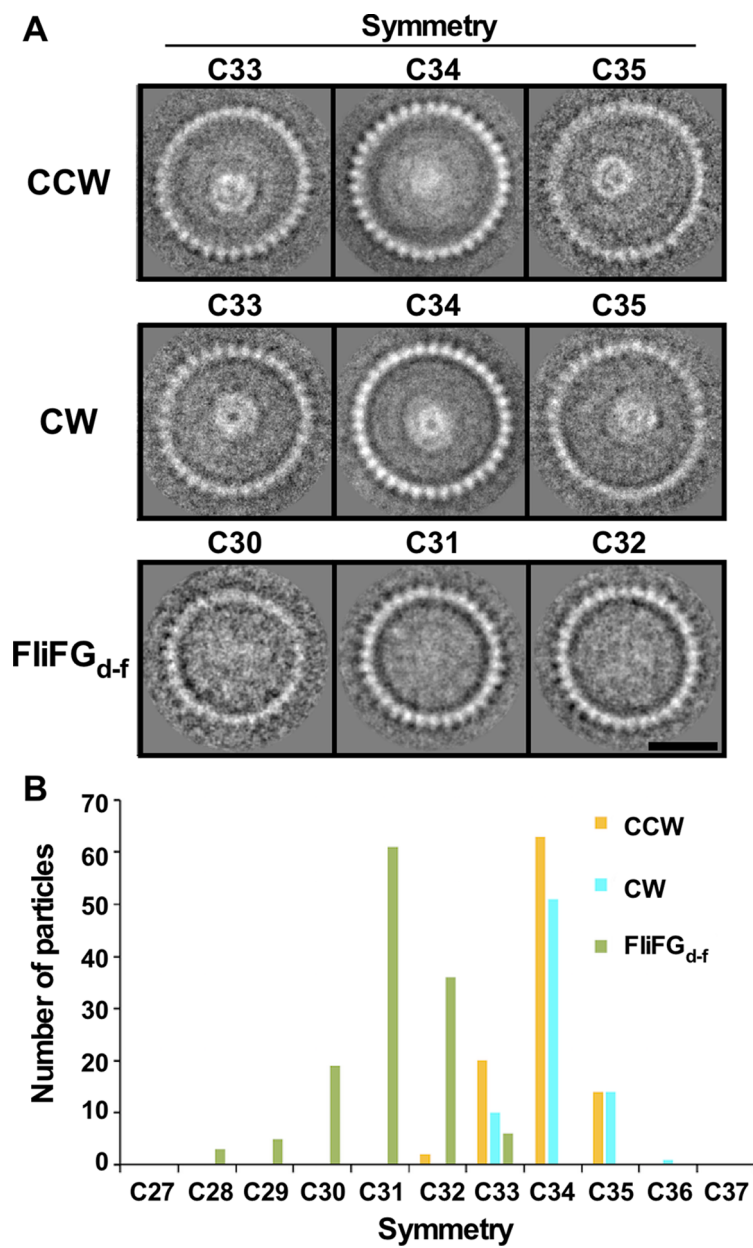

Supplement: FIG S4 [file mBio.00079-19-sf004.pdf]

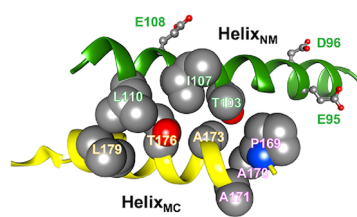

Supplement: FIG S5 [file mBio.00079-19-sf005.pdf]
