# Supplementary material for: Novel Insights into Conformational Rearrangements of the Bacterial Flagellar Switch Complex
Source: mBio. 2019 Apr 2;10(2):e00079-19. doi: 10.1128/mBio.00079-19 (PMC6445934; doi:10.1128/mBio.00079-19)
Supplement: TABLE S1 [file mBio.00079-19-st001.pdf]

Table S1. Rotational speed and torque of the flagellar motor.

Strain	Bead size (μm)	1.5	1.0	0.8	0.6	0.5
wild-type	Speed (Hz)	26 ± 4	71 ± 10	110 ± 20	145 ± 28	174 ± 35
	Torque (pN nm)	1,989 ± 271	1,855 ± 260	1,533 ± 243	1,184 ± 178	854 ± 166
	Number of motors	54	39	49	23	46
$FliFG_{d\text{-}f}$	Speed (Hz)	20 ± 4	54 ± 8	91 ± 15	109 ± 18	120 ± 20
	Torque (pN nm)	1,473 ± 260	1,408 ± 223	1,306 ± 217	892 ± 123	596 ± 84
	Number of motors	33	33	54	24	44
$FliFG_{d\text{-}f}$ FliG(D124Y)	Speed (Hz)	18 ± 5	56 ± 11	88.8 ± 13.9	115 ± 19	135 ± 25
	Torque (pN nm)	1,425 ± 225	1,455 ± 224	1,194 ± 177	912 ± 151	616 ± 126
	Number of motors	15	11	24	24	25
$FliFG_{d\text{-}f}$ FliM(F188L)	Speed (Hz)	18 ± 5	52 ± 8	89 ± 20	123 ± 24	159 ± 30
	Torque (pN nm)	1,377 ± 262	1,338 ± 197	1,220 ± 258	963 ± 153	709 ± 135
	Number of motors	14	11	16	26	26
