# Supplementary material for: Novel Insights into Conformational Rearrangements of the Bacterial Flagellar Switch Complex
Source: mBio. 2019 Apr 2;10(2):e00079-19. doi: 10.1128/mBio.00079-19 (PMC6445934; doi:10.1128/mBio.00079-19)
Supplement: TABLE S2 [file mBio.00079-19-st002.pdf]

Table S2. Speed fluctuations of the flagellar motor.

Strain (Number of motors)		Average ± SD
wild-type (n = 20)	ω_{av} (Hz)	49.0 ± 8.2
	σ_ω	7.6 ± 2.9
	$\sigma_\omega/\omega_{av}$	0.16 ± 0.04
$FliFG_{d\text{-}f}$ (n = 20)	ω_{av} (Hz)	39.9 ± 8.6
	σ_ω	12.3 ± 3.7
	$\sigma_\omega/\omega_{av}$	0.31 ± 0.12
$FliFG_{d\text{-}f}$ FliG(D124Y) (n = 20)	ω_{av} (Hz)	40.1 ± 7.1
	σ_ω	9.4 ± 3.0
	$\sigma_\omega/\omega_{av}$	0.23 ± 0.07
$FliFG_{d\text{-}f}$ FliM(F188L) (n = 20)	ω_{av} (Hz)	40.2 ± 6.4
	σ_ω	6.9 ± 2.1
	$\sigma_\omega/\omega_{av}$	0.17 ± 0.05

The values of the average speeds (ω_{av}) and their standard deviations (σ_ω) were obtained by tracking the position of 1.0 μm bead for 300 seconds. Note: their average motor speeds are slower than those presented in Table S1. Such speed reductions presumably result from a decrease in the energy level of bacterial cells because of much longer-term light illumination.
