# Supplementary material for: Novel Insights into Conformational Rearrangements of the Bacterial Flagellar Switch Complex
Source: mBio. 2019 Apr 2;10(2):e00079-19. doi: 10.1128/mBio.00079-19 (PMC6445934; doi:10.1128/mBio.00079-19)
Supplement: TABLE S3 [file mBio.00079-19-st003.pdf]

Table S3. Strains and plasmids used in this study.

Strain/Plasmid	Relevant characteristics	Reference
E. coli		
BL21(DE3)	Overexpression of proteins	Novagen
Salmonella		
SJW1103	Wild type for motility and chemotaxis	(35)
SJW46	*fliC*(Δ204-292)	(39)
SJW3278	*fliFG*$_{d\text{-}f}$	(9)
HK1003	Δ*flgE* Δ*clpP*::*Cm*	This study
MM046CY	*fliC*(Δ204-292) *cheY*::Tn*10*	This study
MM046mAB	*fliC*(Δ204-292) Δ*motAB*::*tetRA*	This study
MM3278-46	*fliFG*$_{d\text{-}f}$ *fliC*(Δ204-292)	This study
MM3278-46CY	*fliFG*$_{d\text{-}f}$ *fliC*(Δ204-292) *cheY*::Tn*10*	This study
MM3278-46mAB	*fliFG*$_{d\text{-}f}$ *fliC*(Δ204-292) Δ*motAB*::*tetRA*	This study
MM3278-1	*fliFG*$_{d\text{-}f}$ *fliM*(F188L)	This study
MM3278-1-46	*fliFG*$_{d\text{-}f}$ *fliM*(F188L) *fliC*(Δ204-292)	This study
MM3278-1-46CY	*fliFG*$_{d\text{-}f}$ *fliM*(F188L) *fliC*(Δ204-292) *cheY*::Tn*10*	This study
MM3278-1-46mAB	*fliFG*$_{d\text{-}f}$ *fliM*(F188L) *fliC*(Δ204-292) Δ*motAB*::*tetRA*	This study
MM3278-2	*fliFG*$_{d\text{-}f}$ *fliN*(E95G)	This study
MM3278-2-46	*fliFG*$_{d\text{-}f}$ *fliN*(E95G) *fliC*(Δ204-292)	This study
MM3278-2-46CY	*fliFG*$_{d\text{-}f}$ *fliN*(E95G) *fliC*(Δ204-292) *cheY*::Tn*10*	This study
MM3278-5	*fliFG*$_{d\text{-}f}$ *fliM*(I217T)	This study
MM3278-5-46	*fliFG*$_{d\text{-}f}$ *fliM*(I217T) *fliC*(Δ204-292)	This study
MM3278-5-46CY	*fliFG*$_{d\text{-}f}$ *fliM*(I217T) *fliC*(Δ204-292) *cheY*::Tn*10*	This study
MM3278-6	*fliFG*$_{d\text{-}f}$ *fliM*(V186A)	This study
MM3278-6-46	*fliFG*$_{d\text{-}f}$ *fliM*(V186A) *fliC*(Δ204-292)	This study
MM3278-6-46CY	*fliFG*$_{d\text{-}f}$ *fliM*(V186A) *fliC*(Δ204-292) *cheY*::Tn*10*	This study
MM3278-8	*fliFG*$_{d\text{-}f}$ *fliG*(D124Y)	This study
MM3278-8-46	*fliFG*$_{d\text{-}f}$ *fliG*(D124Y) *fliC*(Δ204-292)	This study
MM3278-8-46CY	*fliFG*$_{d\text{-}f}$ *fliG*(D124Y) *fliC*(Δ204-292) *cheY*::Tn*10*	This study
MM3278-8-46mAB	*fliFG*$_{d\text{-}f}$ *fliG*(D124Y) *fliC*(Δ204-292) Δ*motAB*::*tetRA*	This study
TM116	Δ*cheA-cheZ* Δ*fliC*, Δ*clpP*::*Cm*	This study
TM108	*fliG*Δ(P169–A170–A171) Δ*fliC*, Δ*clpP*::*Cm*	This study
TM147	*fliFG*$_{d\text{-}f}$ Δ*clpP*::*Cm*	This study
TM022	Δ*flgE* *fliG*Δ(P169–A170–A171) Δ*clpP*::*Cm*	This study
TM148	Δ*flgE* *fliFG*$_{d\text{-}f}$ Δ*clpP*::*Cm*	This study
Plasmids		
pHS63	pBAD24/MotA+MotB	(28)
